# The Effect of the Surface Topography of High-Speed Steel Cutting Blades Made Using Various Methods on Their Technological and Functional Properties

**DOI:** 10.3390/ma16196442

**Published:** 2023-09-27

**Authors:** Maciej Jan Kupczyk, Jędrzej Komolka, Jerzy Józwik

**Affiliations:** 1 Faculty of Mechanical Engineering, Institute of Mechanical Technology, Poznan University of Technology, Piotrowo 3, 60-695 Poznan, Poland; maciej.kupczyk@put.poznan.pl (M.J.K.); jedrzej.komolka@gmail.com (J.K.); 2 Department in Production Engineering, Faculty of Mechanical Engineering, Lublin University of Technology, Nadbystrzycka 36, 20-618 Lublin, Poland

**Keywords:** high-speed steels, machining, tribological properties, surface oil volume, ranges of justified use

## Abstract

Indication of the proper scope of application of high-speed steels (HSS) produced by conventional methods and powder metallurgy for cutting tool blades requires a thorough assessment of their properties in various cutting conditions. According to common knowledge, the properties of tools made of both types of steel are largely determined by the arrangement of carbide phases. It turns out, however, that the surface morphology of the cutting blades is at least as important for their operational properties. This was already indicated by the preliminary research of the authors of the article, in which it was found that the surface topography significantly affects their durability depending on the applied value of the cutting speed in dry machining conditions and in the presence of a cooling lubricant. As it turned out, contrary to the literature data, blades made of sintered HSS do not have better service properties in all cutting conditions than blades made by conventional methods (e.g., in forging and hot rolling processes). Hence, it was necessary to determine the justified range of use of both types of steel for cutting blades. Cutting blades with a similar chemical composition from conventional high-speed steel and those produced in the powder metallurgy process were tested. Wear curves were determined on the basis of the tests. On this basis, for the assumed value of the blunting index, the tool life of the cutting edges was determined during the cutting of selected structural steel, commonly used for high-strength machine parts. Tests of the cutting ability of the blades were carried out without and with the use of a cooling--lubricating liquid. In order to correctly interpret the obtained results of the wear and durability tests of the cutting edges, verification tests were carried out. A detailed analysis of the research material made it possible to determine the recommended range of applicability of conventional and sintered HSS for the tested case.

## 1. Introduction

According to the source data [1,2,3,4], conventional HSS (high-speed steel) are still quite often applied for the production of cutting blades operating at low and mean cutting speeds. This has not even been affected by the recent development of new materials for the production of cutting tools. This is due to the special properties of high-speed steels. Their main advantages include a relatively low cost of production and higher torsion and bending strength compared to other tool materials. In order to improve the operational properties of cutting blades made of conventional high-speed steels, sintered high-speed steel was gradually introduced [5,6,7,8,9]. Unfortunately, the cost of manufacturing tools from sintered HSS is still relatively considerable and only the large-lot production of these tools is fully profitable [2,4,6,9]. The main profit of using metal ceramics over classical metallurgy is the ability to combine potentially insoluble components that have different chemical bonds and melting points [6,7,8,9,10,11,12,13,14,15,16,17,18,19,20]. Therefore, metal ceramics gives much more freedom in shaping the chemical constitution of the end product.

Indication of the proper scope of application of high-speed steels produced by conventional methods and powder metallurgy for cutting tool blades requires a thorough assessment of their properties in various cutting conditions. According to common knowledge [6,9,17,20], the properties of tools made of both types of steel are largely determined by the arrangement of carbide phases. It turns out, however, that the surface morphology of the cutting blades is at least as significant for their operational properties. This was already indicated by the preliminary research of the authors of the article [2,3,4], in which it was found that the surface topography significantly affects their durability depending on the applied value of the cutting speed in dry machining conditions and using a cooling lubricant. As it turned out, contrary to the literature data, blades made of sintered HSS do not have better service properties in all cutting conditions than blades made by conventional methods (cast and then plastically processed, e.g., in the processes of forging and hot rolling). Hence, it was necessary to determine the justified range of use of both types of steel for cutting blades. This became the main aim of this article.

## 2. Materials and Methods

### 2.1. Cutting Blades

In the tests, two kinds of HSS with similar chemical constitutions were applied to produce multi-edge cutting inserts:

1. HS6-5-2 conventional HSS:
  — Forged;
  — Rolled;
2. PM6-5-2 sintered HSS.

The selection of these steel grades was guided by the fact that these grades are most often applied in industrial practice.

Table 1 presents the approximate chemical constitutions of conventional and sintered HSS according to information from [21] and different standards (DIN, EN, AISI, and PN).

The cutting blades in the form of rectangular plates of conventional HSS were obtained from billets, while the cutting blades of sintered HSS were made from commercial blanks in the form of flat bars. Both kinds of steel were in a softened state before the tool blades were formed from them.

Conventional HSS billets and sintered HSS flats were roughly ground using a surface grinder to obtain the desired thickness of the replaceable cutting inserts. Tool inserts of the SNUN type were made from the prepared blanks using the Agiecut Classic 2 wire EDM machine (manufactured by AGIE SA firm—Losone, Switzerland). After machining, the blades obtained a cuboid shape with the following dimensions: *L* = 9.526 ± 0.07 mm, *m* = 1.645 ± 0.14 mm and a tool tip angle of 90°. Blades with this shape were applied in turning heat-treatable steel, heat-resistant steel, stainless steels, alloy steels and mild steels with low carbon content [3]. Finally, the surfaces of the tool inserts were ground, then polished and degreased. A similar surface roughness was assumed for blades obtained from conventional and sintered high-speed steel, which will enable subsequent correct conclusions.

The assumed hardness of the inserts made of HS6-5-2 and PM6-5-2 steel was obtained after hardening and twice tempering. For the purpose of obtaining a hardness of about 65 HRC, an austenitization temperature of 1190 °C and a tempering temperature of 560 °C and then 540 °C were adopted. Correctly selected tempering temperature allowed for the formation of the phenomenon of secondary hardness. Replaceable cutting inserts of this hardness retain the ability to cut the workpiece at a temperature close to the tempering temperature of the blade material [1,2,3,4,6,7,8,9,11,21].

**Figure 1:**
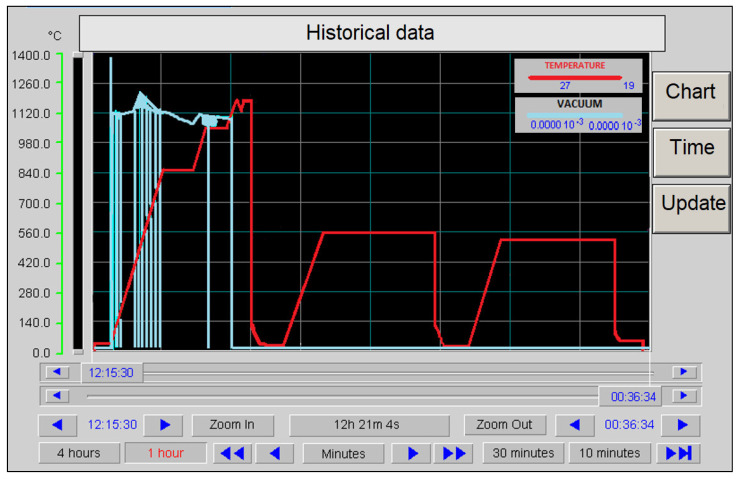
Schematic representation of the heat treatment process of the cutting blades in the vacuum furnace [13] (blue curve—pressure; red curve–temperature).

Considering the low thermal conductivity of high-speed steels, they were heated little by little. The heat treatment process was carried out in a vacuum because of the risk of oxidation and decarburization. Figure 1 schematically presents the course of heat treatment. This process was carried out in a furnace with a high vacuum system Seco/Warwick version 6.0VPT-402/24QHV (manufactured by the E.C.M. Technologies—Grenoble, France).

### 2.2. Workpiece Material

As the workpiece material in the tests, 40HNMA heat-treatable steel with a hardness of 29 ± 2 HRC was used in the form of cylindrical rod with a diameter of ϕ100 mm and a length of 400 mm. It is a grade of constructional alloy steel with high strength (tensile strength is 1080 MPa, and the yield point of this steel is 980 MPa), and it has particularly very good hardenability, applied for thermal improvement for heavily loaded machine elements exposed to impact, torsion and vibrations, where an appropriate impact strength of the product is indicated. Table 2 presents the chemical constitution of 40HNMA steel by PN, AISI, DIN and EN standards.

Contrary to other products for thermal improvement, 40HNMA does not show a tendency to be brittle after tempering, and elements made with it show very good mechanical properties, including strength at cross-sections of approx. 150 mm. 40HNMA steel is a grade intended for hardening in oil at a temperature of 810–840 °C. Process of tempering is carried out at a temperature in the range of 550–650 °C.

Elements made of this material are intended primarily for parts and sub-assembly of turbines that work at temperatures up to ~450 °C, as aviation material for loaded parts of aircraft, axles and rods, and elements of internal combustion engines.

This steel is generally used in special machine elements such as cranked rods, connecting shafts, propeller hubs, control elements, compressor elements, single-disc rotors, pump elements, press elements, multi-disc rotors, large non-carburized gears, steam turbine drums, screws with large diameters, steam turbine discs, large diameter screws, steam turbine shafts and control parts.

## 3. Functional Property Tests

The evaluation of the functional properties of cutting blades made of conventional and sintered HSS was made on the basis of wear and durability tests of the blades in the machining of 40HNMA structural steel, hardened and tempered to a hardness of 29 ± 2 HRC.

The replaceable cutting blades made in the previously mentioned manner were fixed in the holder type hR 110.16–220. After mounting the cutting blades in the turning shank, the geometry given below was received:

- Entering angle *κ_r_* = 75°;
- Relief angle α_o_ = 6°;
- Rake angle *γ*_o_ = −6°;
- Inclination angle of the main edge *λ_s_* = −6°;
- Point angle *ε_r_* = 90°.

The following cutting conditions were adopted:

- Feed *f* = 0.2 mm/rev;
- Depth of cut *a_p_* = 0.75 mm;
- Cutting speed *v_c_*_1_ = 34; *v_c_*_2_ = 43; *v_c_*_3_ = 60 m/min;
- “Dry” machining or machining in the presence of a cooling—lubricating emulsion;
- Workpiece material made of 40HNMA steel.

During cutting, a partially synthetic 6% emulsion S455N Statoil Toolway of Norwegian production was used as a cooling and lubricating liquid.

Routinely, prior to the use of replaceable indexable inserts in the cutting tests, the cutting edges and the condition of the flank and rake surfaces were checked. No defects in the form of cracks or chips were observed on any of the tiles submitted for testing.

**Figure 2:**
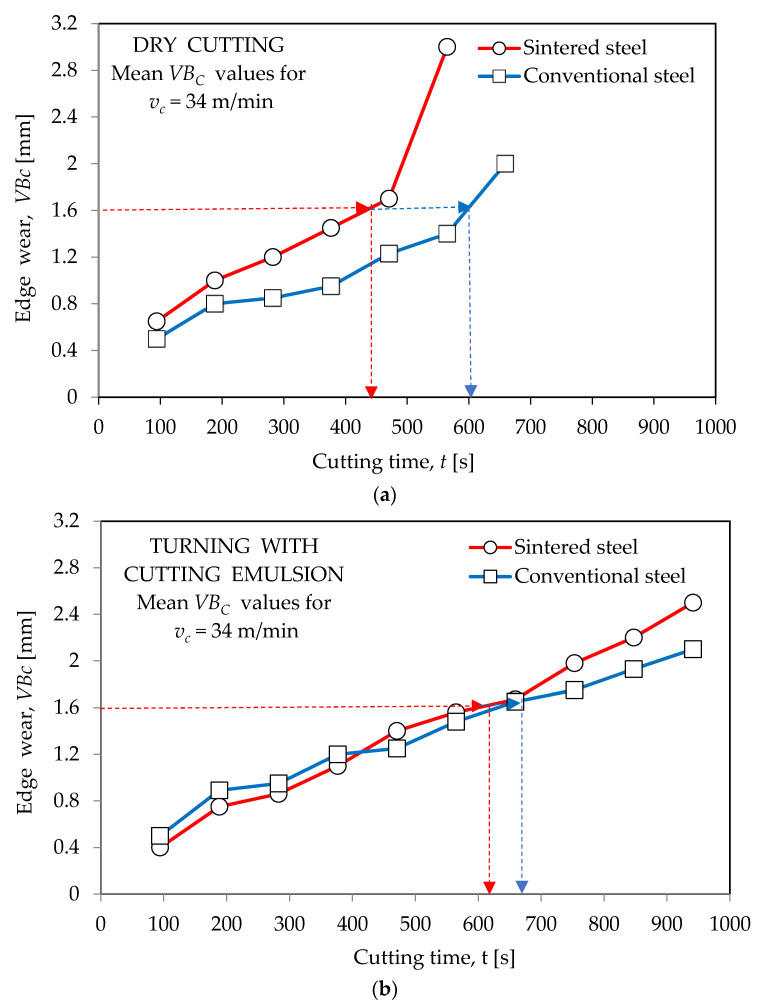
The course of wear for blades with conventional steel HS6-5-2 and sintered steel PM6-5-2 obtained during turning of 40HNMA steel: (**a**) “dry” and (**b**) with cooling and lubricating liquid for *v_c_*_1_ = 34 m/min.

**Figure 3:**
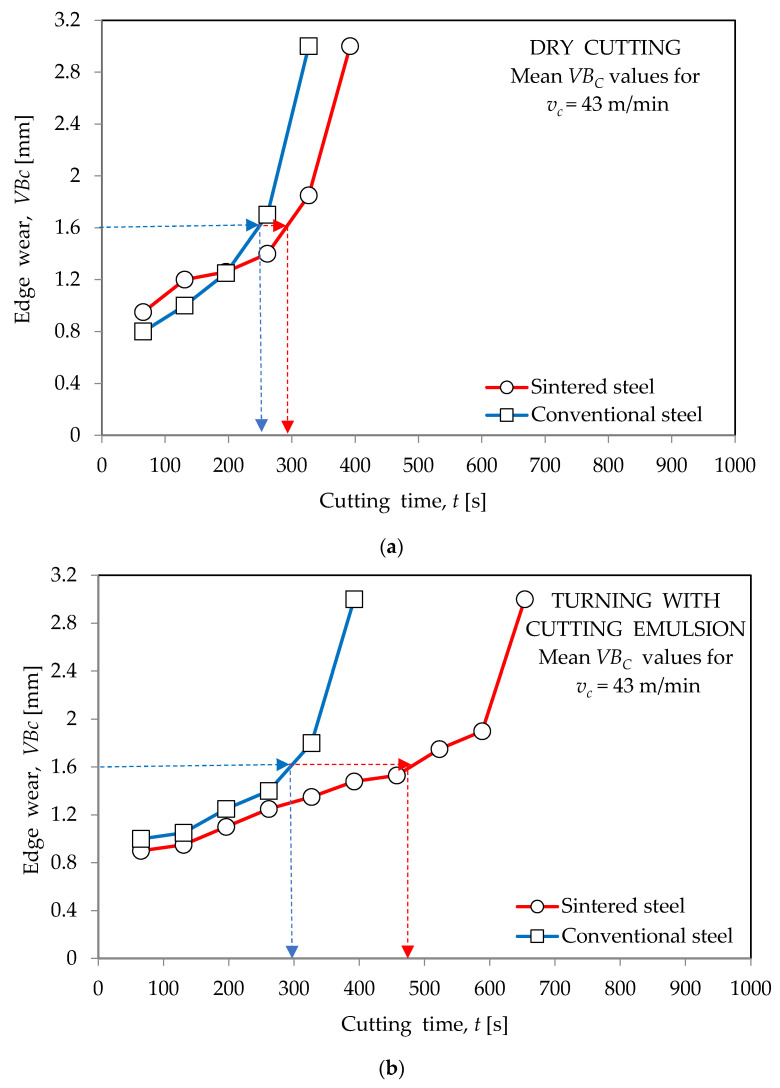
The course of wear for blades with conventional steel HS6-5-2 and sintered steel PM6-5-2 obtained during turning of 40HNMA steel: (**a**) “dry” and (**b**) with cooling and lubricating liquid for *v_c_*_2_ = 43 m/min.

**Figure 4:**
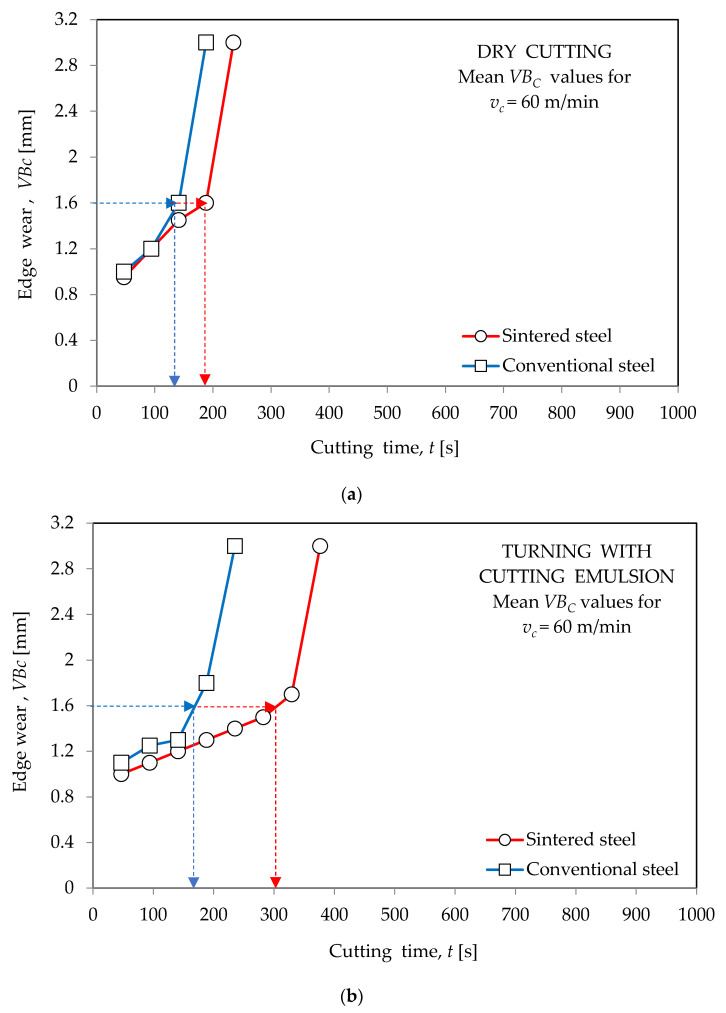
Wear curves of blades made of conventional steel HS6-5-2 and sintered steel PM6-5-2 obtained during turning of 40HNMA steel: (**a**) “dry” machining; (**b**) with cooling and lubricating liquid for *v_c_*_3_ = 60 m/min.

Figure 2, Figure 3 and Figure 4 show the wear curves of the cutting blades prepared on the basis of tests of blades made of both types of high-speed steel in “dry” working conditions and with the use of a cooling--lubricating emulsion. The test results, as previously stated, apply to cutting inserts made of conventional HSS (HS6-5-2) and sintered HSS (PM6-5-2).

The determined wear curves were used to define the durability of the cutting blades. In accordance with the principles adopted in the investigations of the utility properties of cutting blades, durability was determined as the time of cutting until the assumed value of the dulling index was reached. Based on the course of the wear curves received during the entrance tests and taking into account data from other publications (including the relevant standards), a value of *VB_c_* = 1.6 mm, which is the value of the indicator of the edge blunting, which is the width of the corner blunting band from the side of the flank face, was used. The *VB_c_* value was measured using a bench microscope.

**Figure 5:**
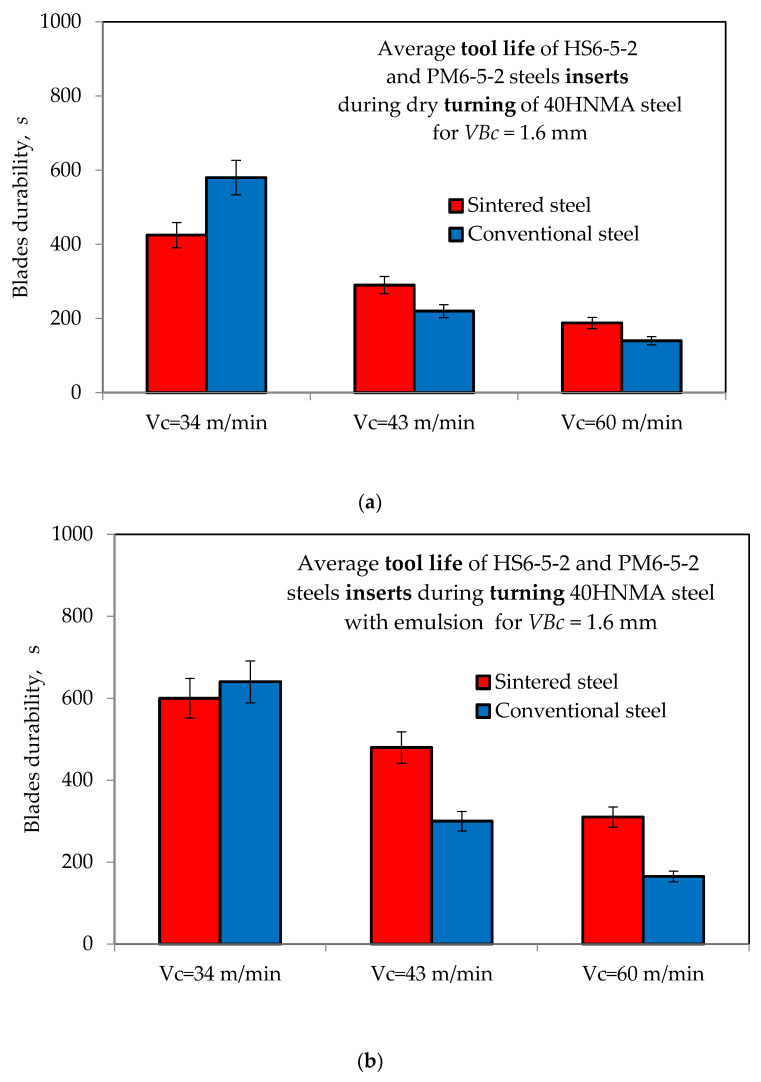
Comparison of the results of the average durability of blades with conventional and sintered high-speed steel during machining of 40HNMA steel (**a**) “dry” and (**b**) with a cooling--lubricating emulsion.

The results of the tool life tests of cutting blades with conventional and sintered HSS obtained when machining 40HNMA steel during a “dry” operation and with the use of a cooling--lubricating emulsion are shown in Figure 5.

## 4. Verification Tests

The purpose of the verification tests was to determine the reasons for the different behavior of tool edges with conventional and sintered HSS when machining 40HNMA structural steel in “dry” working conditions and using a cooling--lubricating emulsion.

First, the hardness of conventional and sintered high-speed steel was determined. In the Vickers hardness attempt, a Picodentor HM50 hardness device by Fischer (Sindelfingen) was applied. According to the applicable standard, hardness was estimated using a penetrator load of 30 kG for 20 s. The hardness of each replaceable cutting plate was measured five times, taking into account the condition that the spacing of the indentations was not less than three times their diagonal. Based on the obtained measurement results, it was found that the hardness of the sintered steel blades is almost 5% greater than the hardness of conventional HSS. The average value for sintered steel was 1030.5 HV30, and that for conventional steel 982.3 HV30. The measurement results were consistent with Sandvick data [21].

**Figure 6:**
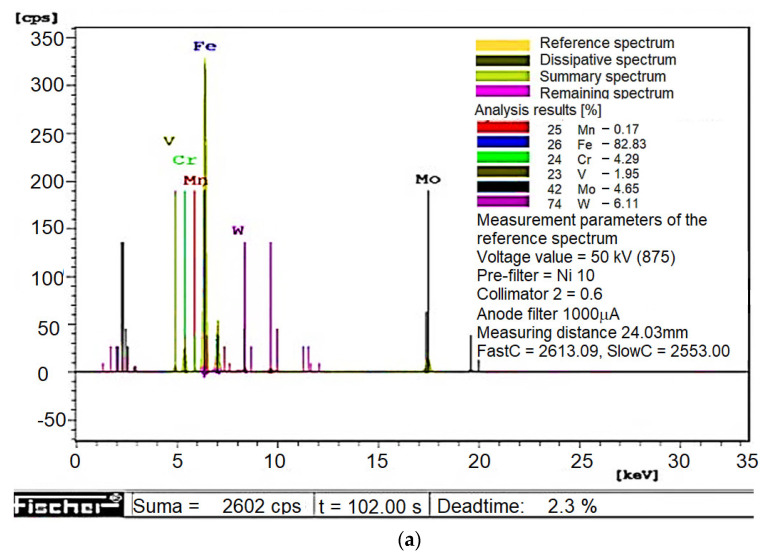

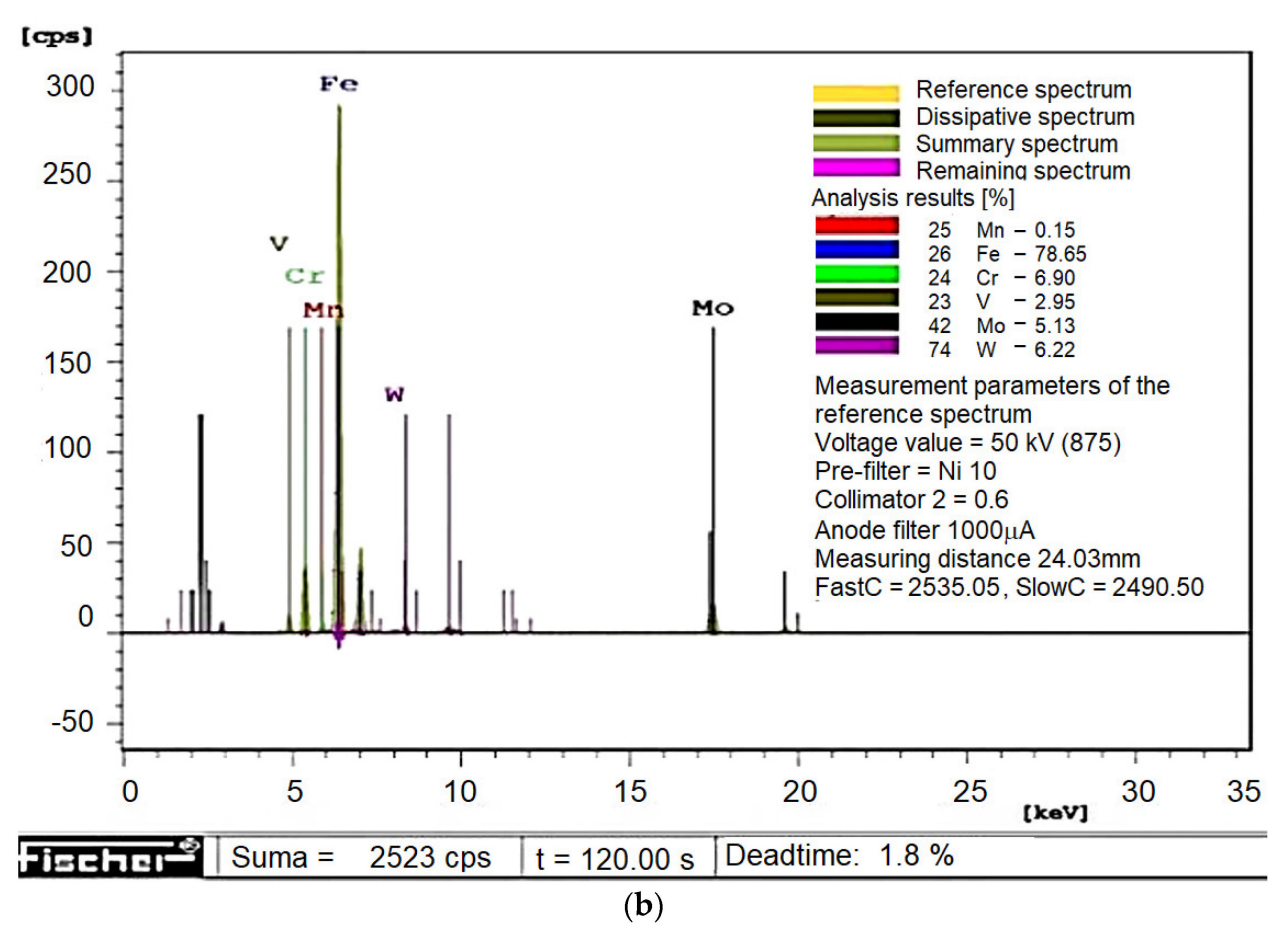
Selected microanalysis images of tool inserts made of (**a**) conventional and (**b**) sintered steels.

The actual chemical constitution of high-speed steels was determined by applying an X-ray fluorescence spectrometer-XD-SD (manufactured by Fischer, Sindelfingen, Germany) [3]. Exemplary test results of the chemical composition of conventional and sintered HSS are shown in Figure 6. Eight measurements were taken. The mean values of the content of alloying elements did not differ meaningfully from the values included in Table 2 for a number of degrees of freedom of 7 and an assumed significance level of *α* = 0.05.

**Figure 7:**
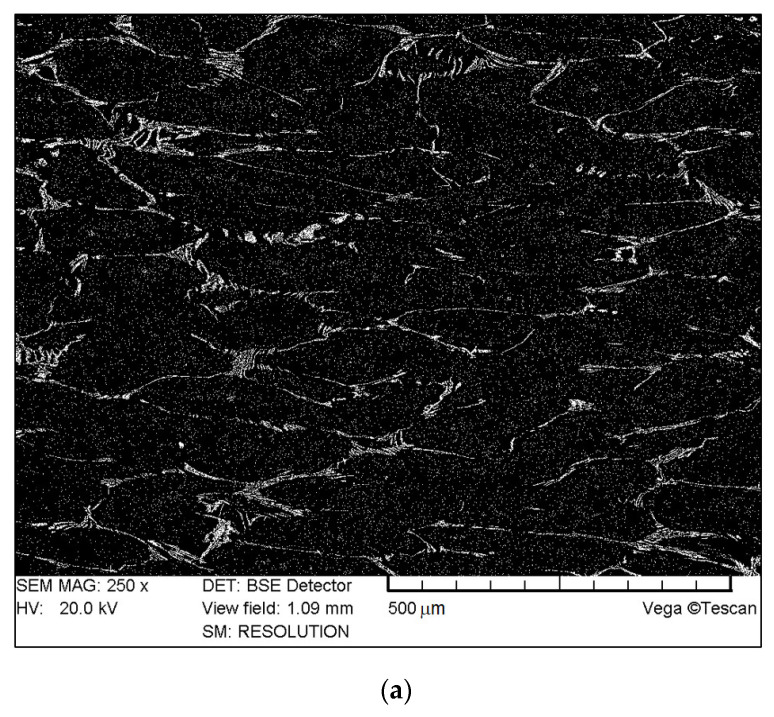

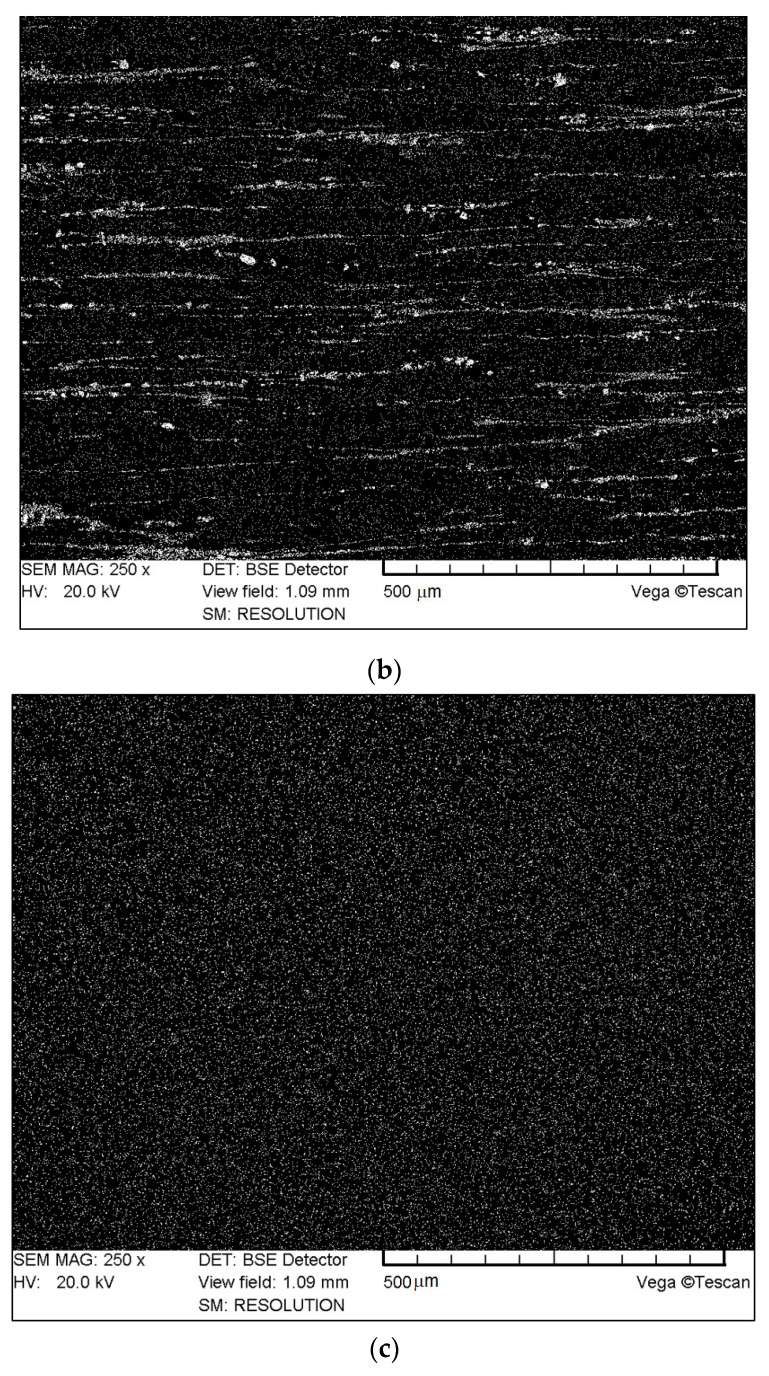
Example images (created using reflected electrons) of microsections of cutting blades made of (**a**) hot-rolled steel, (**b**) forged steel and (**c**) sintered steel.

A Tescan Vega 5135 scanning microscope (Tescan, Brno, Czech Republic) was applied to take pictures of the surfaces of conventional and sintered replaceable cutting blades made of HSS [4] (Figure 7).

BSE microscopic images of metallographic microsections revealed an even distribution of carbides in the matrix in the case of sintered steels.

In the case of conventional HSS (forged or hot-rolled), the BSE images confirm that the carbides are not evenly distributed and form local agglomerates in the shape of bands. This is typical of HSS after stretch forging, rolling or stretch forging with indirect upsetting.

**Figure 8:**
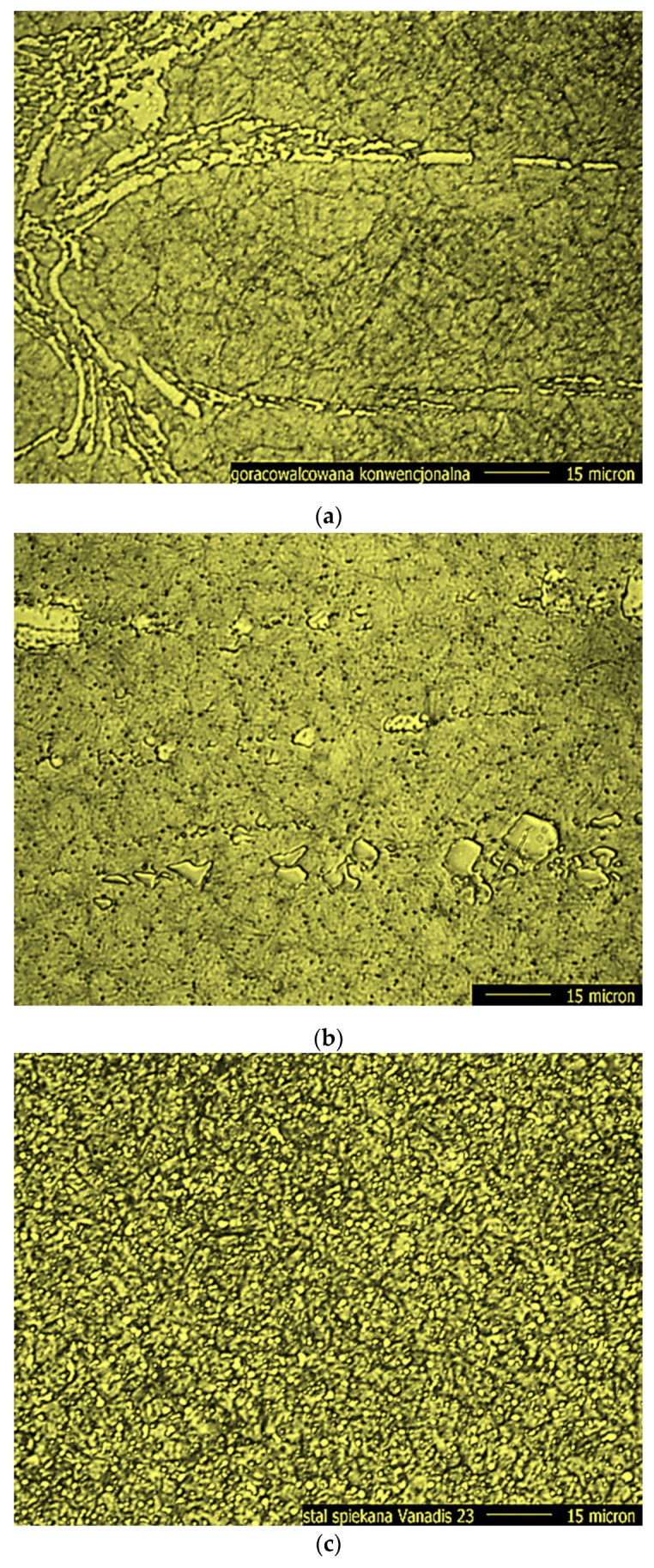
Selected images of secondary electrons (SE) showing the surface morphology of HSS cutting blades with (**a**) hot-rolled, (**b**) forged and (**c**) sintered steels.

The Neophot 32 toolmaker’s microscope (Carl Zeiss—Jena, Germany) was also used to observe the surfaces of the replaceable tool blades with conventional and sintered HSS [3]. With its help, a series of photos of the surfaces of replaceable cutting inserts were taken (Figure 8).

**Figure 9:**
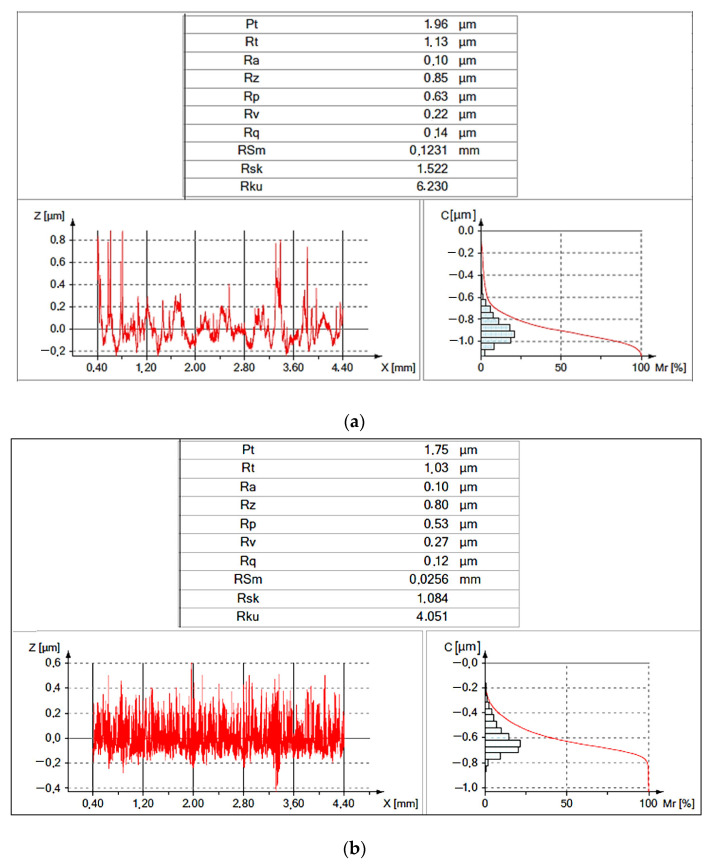
Results of selected parameters values of surface roughness of cutting edges with HSS: (**a**) conventional and (**b**) sintered.

Microscopic images of the surface of the cutting blades revealed a clear diversification in the surface topography of conventional and sintered HSS. The surfaces of conventional hot-rolled and conventional forged steels consist of grains that “blend” together to form a smooth surface. The surface of steel produced by powder metallurgy has a different topography because it is made of sharp-edged grains. Despite the fact that the surface topography of the tested samples made of conventional and sintered steels is different, they have equal surface roughness, i.e., *Ra* = 0.1 μm (Figure 9).

**Figure 10:**
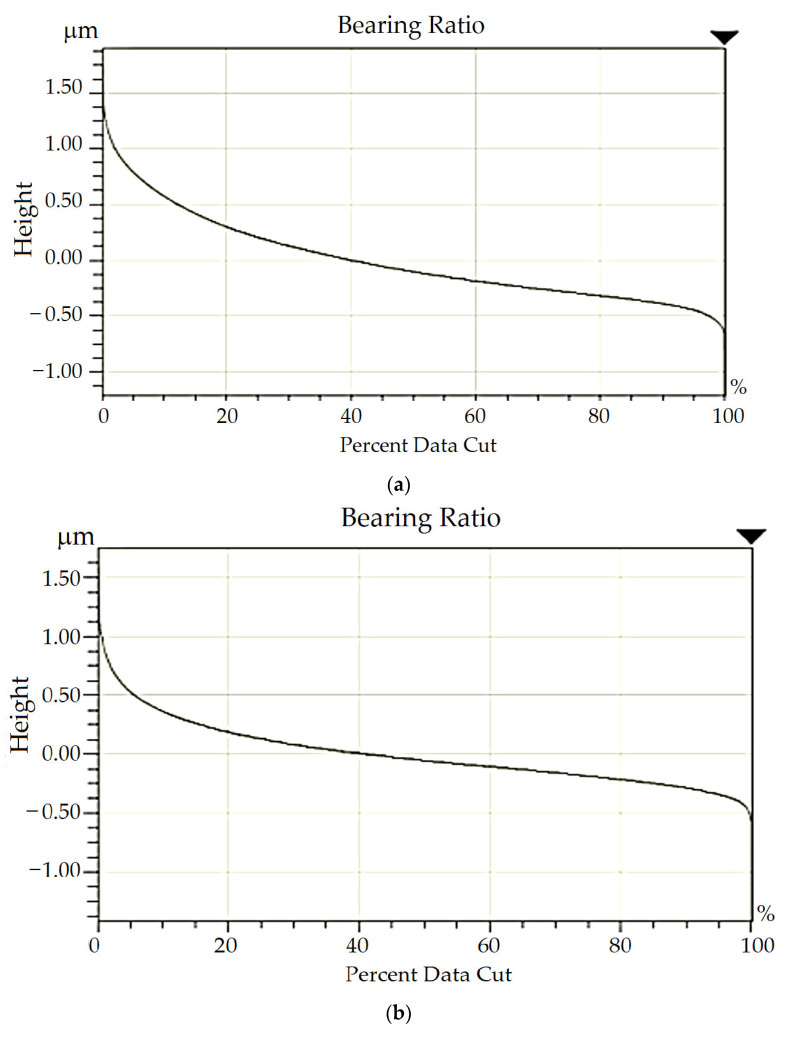

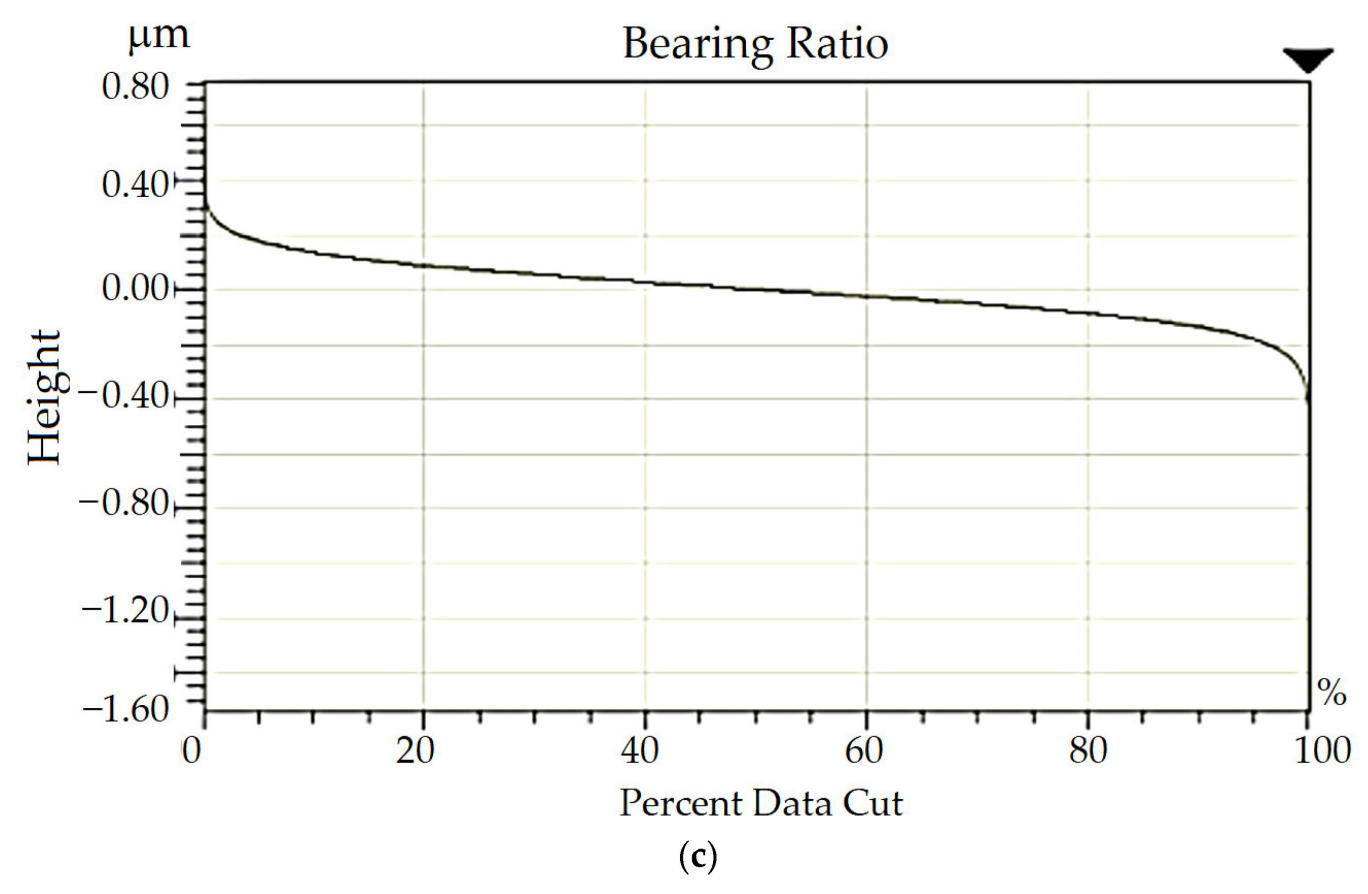
Chosen data on surface morphology taking into account Abbott–Firestone curves for tool blades made of (**a**) hot rolled, statistics: *M_r_*_1_ = 22.12%, *M_r_*_2_ =95.98%, *R_vk_* = 142.13 nm, (**b**) forged, statistics: *M_r_*_1_ = 17.31%, *M_r_*_2_ =93.79%, *R_vk_* = 137.88 nm and (**c**) sintered steels, statistics: *M_r_*_1_ = 9.98%, *M_r_*_2_ =89.44%, *R_vk_* = 136.47 nm.

More detailed data of the surface topography, taking into account the Abbott–Firestone curves for the cutting inserts with HSS used, are shown in Figure 10.

Based on the measured parameters of the surface morphology, the so-called surface oil volume of cutting blades made of conventional forged, conventional hot-rolled and sintered high-speed steel was determined. The value of the surface oil volume was calculated from the following relationship [3]:

*V_o_* = *R_vk_*(100 − *M_r_*_2_)/2.000 [mm^3^/cm^2^], (1)

where *V_o_* is the surface oil volume, *R_vk_* is the surface recess and *M_r_*_2_ is the material share for the lower limit of the roughness profile core.

The *R_vk_* parameter characterizes the recess of the surface. It is a quantity describing the ability of the working surface of the cutting blade to retain lubricant in existing recesses. The parameter *M_r_*_2_ defines the value of the material share for the lower boundary of the core roughness profile, while *M_r_*_1_ is the value of the material share for the upper boundary of the core roughness profile. The quantities characterized above provide data about surface irregularities, which considerably influence the behavior of the cutting blades during the turning process.

**Figure 11:**
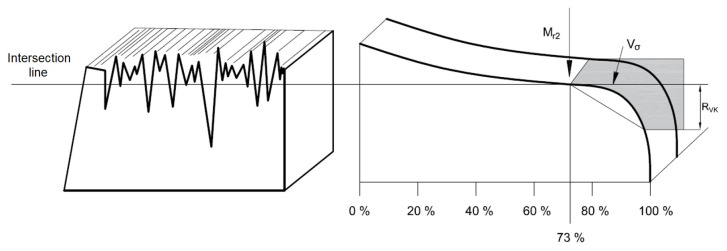
Surface of the material with parameters relevant to determining the surface oil volume, where *M_r_*_2_ is the value of the material share for the lower boundary of the roughness core, *V_o_* is the surface oil volume and *R_vk_* is the surface recess value [22].

Abbott curves describe the shape of the surface as a function of depth relative to the intersection line (Figure 11).

**Figure 12:**
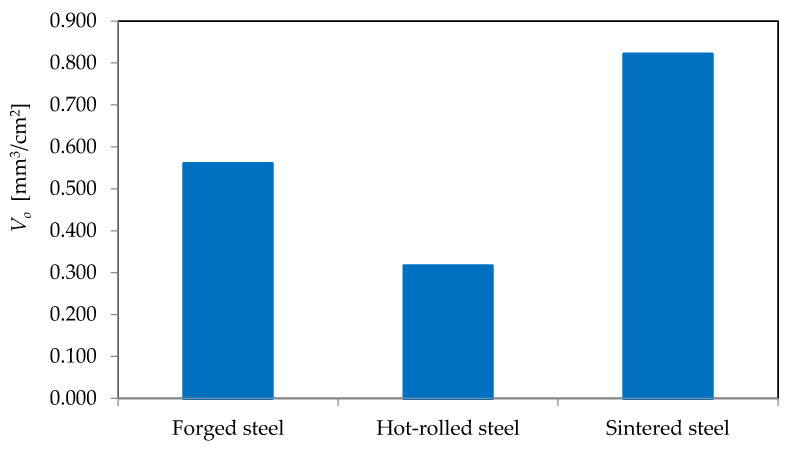
Average values of surface oil volume for applied cutting blades made of high-speed steel.

Figure 12 presents the results of the mean value of the surface oil volume for tool blades with hot-rolled, forged and sintered HSS.

Based on conducted research, it was found that despite the same roughness of the surface (amounting to *Ra* = 0.1 μm) of the tool blades with conventional steel (forged and hot-rolled steel) and sintered HSS, the tool blades differ significantly in terms of surface volume oil.

The surface oil volume of sintered HSS is twice that of conventional forged HSS and nearly three times that of conventional hot-rolled HSS.

For a full interpretation of the tests of the service properties of the replaceable cutting inserts obtained during the machining of 40HNMA steel, further tests were carried out while including the coefficient of dry friction and the coefficient of friction by using a cooling--lubricating emulsion.

The following conditions were adopted during the tribological tests:
– Sample:• Conventional steel (HS6-5-2); • Sintered steel (PM6-5-2);– Counter-sample:• 40HNMA;– Circumferential speed of the counter-sample:  • 27.5 m/min;– Load F (N):• 300, 400, 500 and 600 N;– Time t (s):• 1200 s during dry friction; • 2400 s during friction in the presence of coolant;– Friction:• Dry, • In the presence of a cooling--lubricating emulsion.

**Figure 13:**
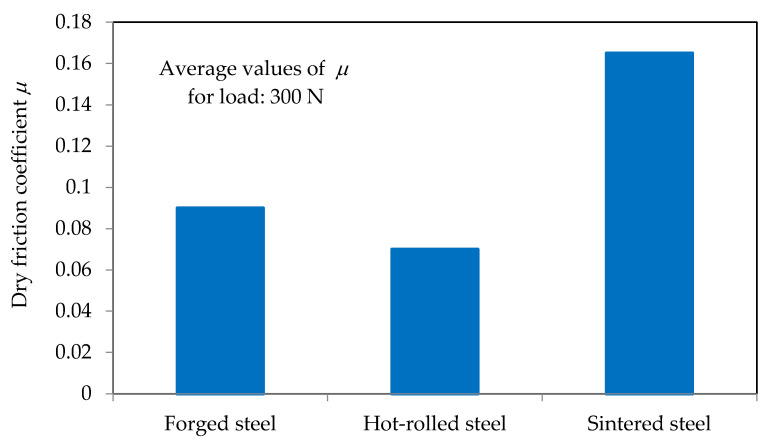
Results of dry friction test.

Figure 13 presents the obtained values of the dry friction coefficient.

The obtained results of the friction tests fully confirmed the results of the tests of functional properties (i.e., durability) carried out during machining without the use of lubrication. The coefficient of friction measurements confirmed also the conclusions resulting from the microscopic images (higher coefficient of friction for the surface of the sintered material made of sharp-edged grains and lower coefficient of friction for the surface consisting of rounded grains forming a continuous surface in the case of conventional HSS).

**Figure 14:**
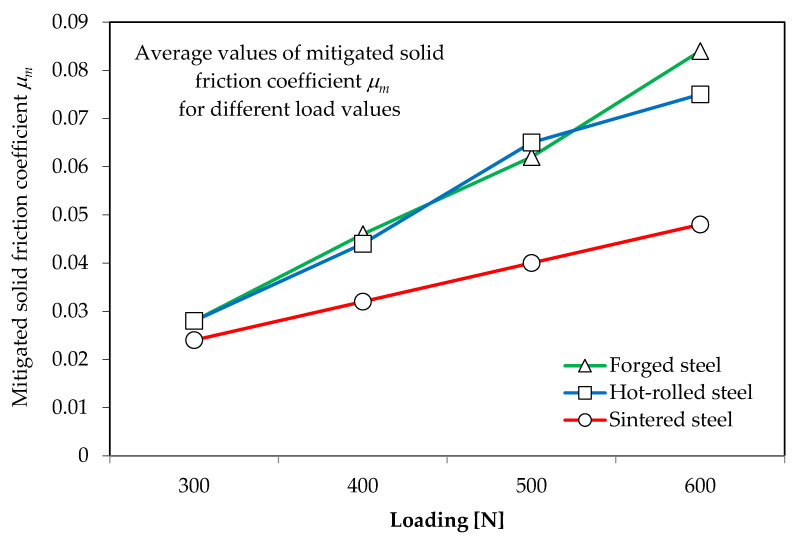
Coefficient of friction in the presence of a cooling--lubricating emulsion for different load values.

The results of the friction coefficient in the presence of a cooling--lubricating emulsion for different load values are shown in Figure 14.

**Figure 15:**
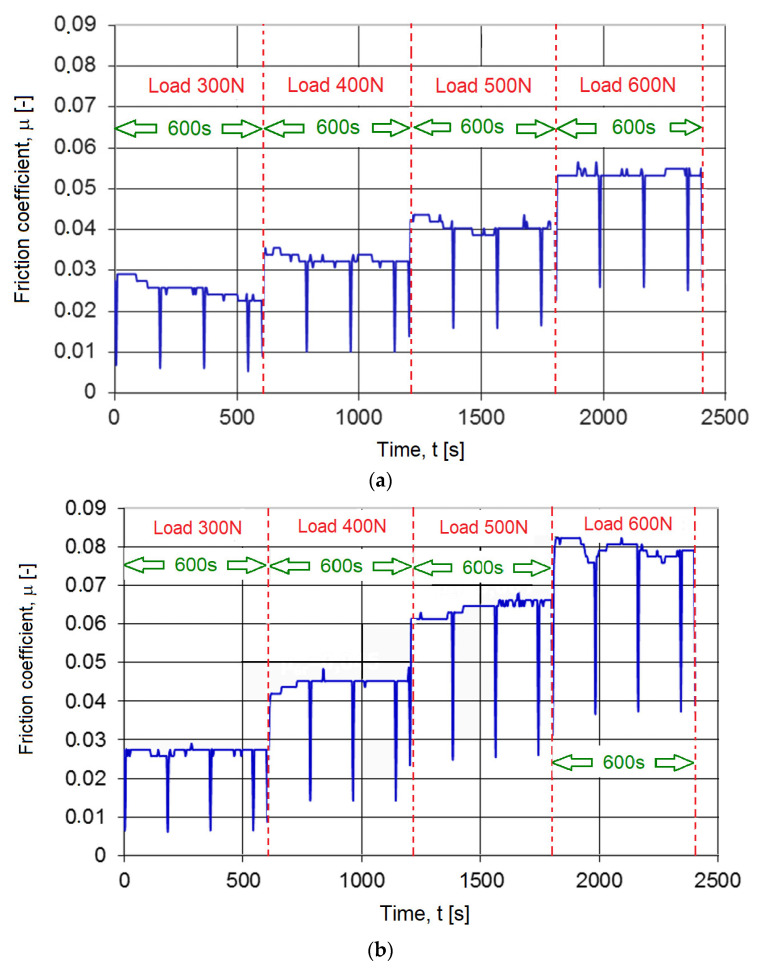

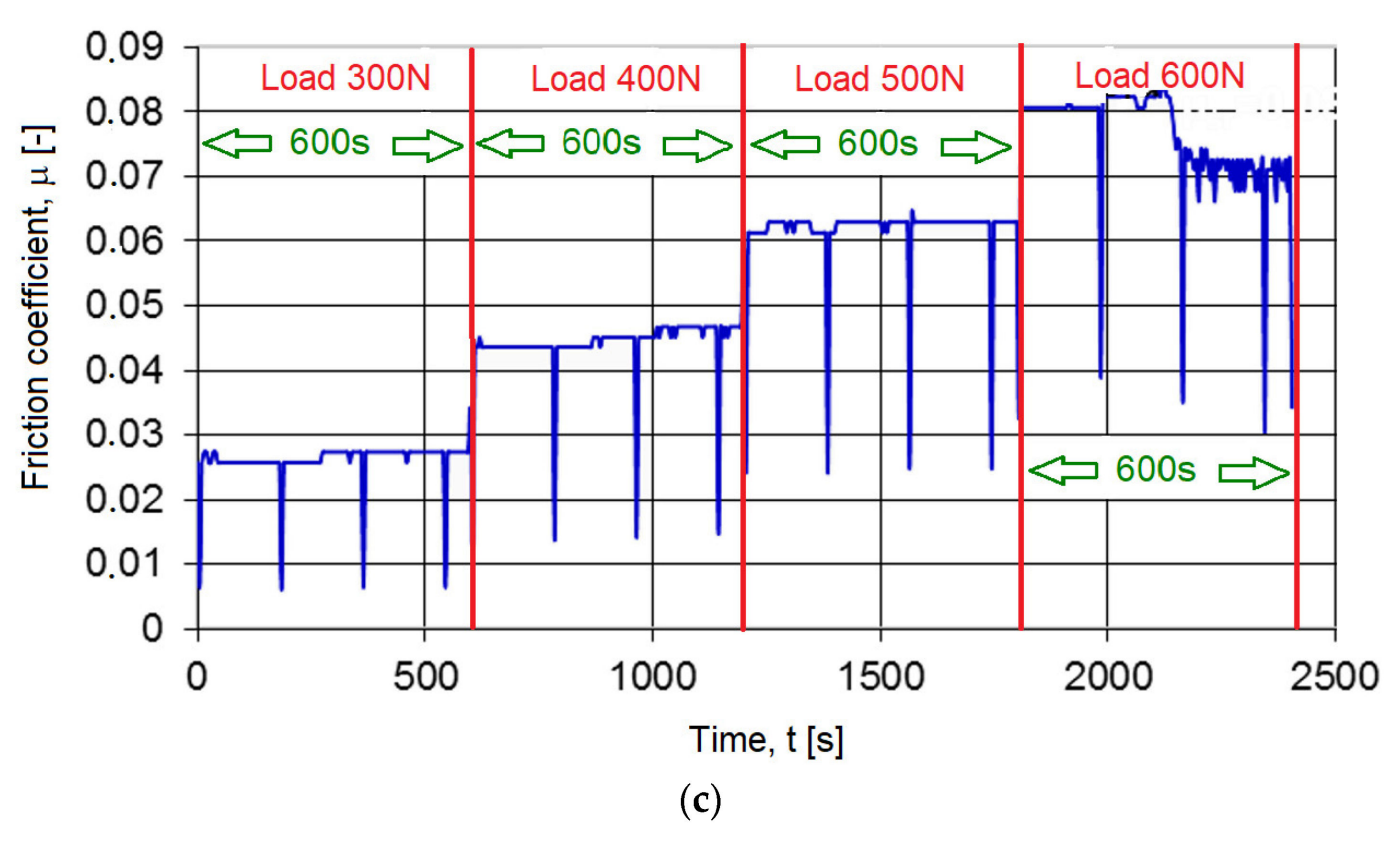
The value of the friction coefficient in the presence of a cooling--lubricating emulsion versus time for subsequent load values for cutting blades made of HSS materials: (**a**) sintered, PM6-5-2; (**b**) conventional forged, HS6-5-2; (**c**) conventional hot-rolled, HS6-5-2.

No change in the value of the friction coefficient in the presence of coolant was observed as a function of time (0–2400 s) at constant load (300, 400, 500 or 600 N) for all tested high-speed steels. In the study of the coefficient of dry friction as a function of time (0–1200 s), the value of *μ* for sintered high-speed steel increased slightly after 1200 s (by about 8%), which can be considered negligible. Figure 15 presents sample waveforms of the value of the friction coefficient in the presence of a cooling--lubricating emulsion versus time for subsequent load values for cutting blades made of different HSS materials. The friction coefficient during tests for the sintered material PM6-5-2 is presented in Figure 15a, that for the conventional forged HS6-5-2 material is presented in Figure 15b and that for the conventional hot-rolled material HS6-5-2 is presented in Figure 15c.

The presented characteristics (Figure 15) confirmed the lack of significance of changes in the friction coefficient at constant load in the tested time window of 600 s. In the case of conventional steels, it remained at the same level all the time. To sum up, no significant effect of time on the value of the friction coefficient of HSS samples against the workpiece was observed in the examined case.

## 5. Conclusions

The conducted research, through a comprehensive assessment of the functional properties of cutting blades made of conventional and sintered high-speed steel, allowed us to indicate the ranges of justified applicability for both types of tool materials.

The test results showed that tool blades made of sintered HSS during machining of 40HNMA steel in the presence of a cooling--lubricating emulsion show much greater durability than blades made of conventional HSS (1.67 times for *v_c_* = 43 m/min and 1.76 times for *v_c_* = 60 m/min) due to the larger surface oil volume, the property of which significantly reduces the value of the coefficient of friction between the cutting edge and the workpiece. A relationship was also observed, indicating the justified use of sintered HSS at higher values of cutting speed.

In the conditions of machining without a cutting fluid, cutting blades made of conventional HSS, for the lowest value of the cutting speed used in the tests (*v_c_* = 34 m/min), are characterized by greater durability than blades made of sintered steel. This is due to the more favorable surface topography, i.e., the lack of sharp-edged grains, as stated in the verification tests. This type of shaping of the surface of the cutting blades made of conventional HSS resulted in a lower value of the coefficient of dry friction against the workpiece than that of the sintered HSS.

The conducted research confirmed the literature data that replaceable tool blades made of sintered steel are characterized by a more even distribution of carbides in the matrix. In the case of these steels, there are no unfavorable carbide bands that occur after the forging or rolling processes of conventional HSS. As the research has shown, despite this advantageous feature, sintered high-speed steels, due to the unfavorable surface topography, are not recommended for “dry” work at lower values of the cutting speed. Under these conditions, they are characterized by worse durability than conventional high-speed steels.

At the same time, the conducted research allowed for the above statements to be generalized due to similar observations made when processing other structural steel [23].

## Figures and Tables

**Table 1:** Chemical constitution of conventional (HS6-5-2) and sintered (PM6-5-2) steels.

Specification	Chemical Constitution (%)					
	C	W	Mo	V	Cr	Mn
Conventional steel HS6-5-2	Standards: PN-EN–HS6-5-2 (SW7M), EN–HS6-5-2, AISI–M2, DIN–1.3343					
	0.8–0.84	5.9–6.7	4.7–5.2	1.7–2.0	4.2	0.4
Sintered steel PM6-5-2	0.86–1.0	6.0–6.4	5.0	1.9–2.0	4.2–4.5	0.3

**Table 2:** Marking and chemical constitution of the 40HNMA steel (by PN-EN ISO 4597).

**Counterpart (by Standards)**							
PN		DIN		EN		AISI	
40HNMA		1.6565		40NiCrMo_4_		4340	
**Chemical Constitution (%)**							
C	Si	Mn	Cr	Mo	Ni	S	P
0.37–0.44	0.17–0.37	0.50–0.80	0.60–0.90	0.15–0.25	1.25–1.65	˂0.025	˂0.03
